# Role of Bassoon and Piccolo in Assembly and Molecular Organization of the Active Zone

**DOI:** 10.3389/fnsyn.2015.00019

**Published:** 2016-01-12

**Authors:** Eckart D. Gundelfinger, Carsten Reissner, Craig C. Garner

**Affiliations:** ^1^Department Neurochemistry and Molecular Biology, Leibniz Institute for NeurobiologyMagdeburg, Germany; ^2^Center for Behavioral Brain SciencesMagdeburg, Germany; ^3^Medical Faculty, Otto von Guericke UniversityMagdeburg, Germany; ^4^German Center for Neurodegenerative Diseases (DZNE) Site MagdeburgMagdeburg, Germany; ^5^Institute of Anatomy and Molecular Neurobiology, Westfälische Wilhelms UniversityMünster, Germany; ^6^German Center for Neurodegenerative Diseases (DZNE) Site BerlinBerlin, Germany; ^7^Charité Medical UniversityBerlin, Germany

**Keywords:** Bassoon, Piccolo, Aczonin, cytomatrix at the active zone, neurotransmitter release, synapto-nuclear signaling, actin dynamics, synaptic vesicle

## Abstract

Bassoon and Piccolo are two very large scaffolding proteins of the cytomatrix assembled at the active zone (CAZ) where neurotransmitter is released. They share regions of high sequence similarity distributed along their entire length and seem to share both overlapping and distinct functions in organizing the CAZ. Here, we survey our present knowledge on protein-protein interactions and recent progress in understanding of molecular functions of these two giant proteins. These include roles in the assembly of active zones (AZ), the localization of voltage-gated Ca^2+^ channels (VGCCs) in the vicinity of release sites, synaptic vesicle (SV) priming and in the case of Piccolo, a role in the dynamic assembly of the actin cytoskeleton. Piccolo and Bassoon are also important for the maintenance of presynaptic structure and function, as well as for the assembly of CAZ specializations such as synaptic ribbons. Recent findings suggest that they are also involved in the regulation activity-dependent communication between presynaptic boutons and the neuronal nucleus. Together these observations suggest that Bassoon and Piccolo use their modular structure to organize super-molecular complexes essential for various aspects of presynaptic function.

## Introduction

**Figure 1:**
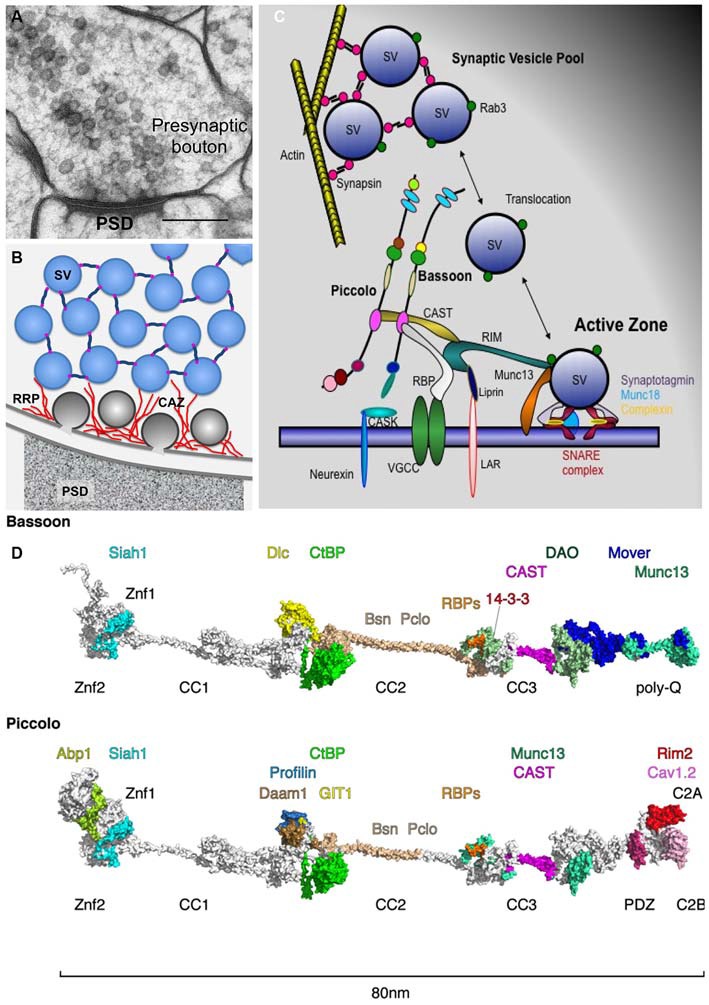
**Piccolo and Bassoon at presynaptic active zones (AZ). (A)** Cryo-electron micrograph of an excitatory synapse from rat brain (originally published in Rostaing et al., 2006; size bar, 200 nm). **(B)** Schematic organization of SVs within presynaptic boutons with SVs in the reserve pool tethered together via synapsin and those in the docked pool embedded in the CAZ (red filaments). **(C)** Schematic of CAZ molecules directing the clustering, translocation, docking, positional and molecular priming and fusion of SVs. **(D)** *In-silico* modeling of Bassoon and Piccolo structures with docking sites for binding partners color coded. The structures include X-ray and NMR data for Znf [protein data bank (Berman et al., 2000), PDB entry: 1ZBD], PDZ (1UJD), coiled-coil (3QH9 and Gruber and Lupas, 2003) and C2 (1RH8, 5CCG) domains. Piccolo C-terminus has been arranged similar to synaptotagmin (Zhou et al., 2015). Most of the remaining parts contain proline and glycine residues that prevent persistent folding but build compact distorted domains (Quiroz and Chilkoti, 2015), while the homologs coiled-coil helices elongate both proteins to about 80 nm. These domains were modeled *ab initio* using threading (Kelley and Sternberg, 2009) and BLAST routines (Sauder and Dunbrack, 2000) and visualized using PyMOL (https://www.pymol.org). For further details compare Table 1. Abbreviations: PSD, postsynaptic density; SV, synaptic vesicle; RRP, release-ready SV pool; CAZ, cytomatrix assembled at the active zone.

Synapses are sophisticated cellular devices designed for the efficient and rapid communication between neurons via the regulated release of neurotransmitter substances from presynaptic boutons and their detection by postsynaptic receptor systems. The release of neurotransmitters is mediated by the recruitment and fusion of synaptic vesicles (SVs) at specialized regions of the presynaptic plasma membrane called active zones (AZ). The molecular and ultra-structural characterization of these release sites revealed that they are composed of a dense cytomatrix assembled at the AZ (CAZ). This matrix is organized by a small number of multi-domain proteins, including Munc13s, Rab3-interacting molecules (RIMs), RIM-binding proteins (RBPs), Liprins-α, ELKS2/CAST as well as the two large scaffolding proteins Bassoon and Piccolo (aka Aczonin; Figure 1) (Garner et al., 2000; Schoch and Gundelfinger, 2006; Gundelfinger and Fejtova, 2012; Südhof, 2012; Ackermann et al., 2015). This review will focus on the contributions of Bassoon and Piccolo to the molecular and functional organization of the CAZ.

Bassoon and Piccolo were originally discovered in a screen designed to identify structural components of rat brain synaptic junctions (Cases-Langhoff et al., 1996; Langnaese et al., 1996). Their molecular characterization revealed that they are structurally related multi-domain proteins including ten highly conserved regions, Piccolo-Bassoon homology domains (tom Dieck et al., 1998; Wang et al., 1999; Fenster et al., 2000). Initially these molecules were thought to be vertebrate specific, however, structurally more distantly related versions are also found in invertebrates, i.e., Fife and Bruchpilot (Wagh et al., 2006; Sigrist and Schmitz, 2011; Bruckner et al., 2012; Ackermann et al., 2015) that may serve similar functions in the CAZ. In vertebrates, Piccolo and Bassoon are selectively localized to the AZs of central and peripheral synapses as well as peptide/protein-secreting cells of the endocrine system (Cases-Langhoff et al., 1996; tom Dieck et al., 1998, 2005; Brandstatter et al., 1999; Richter et al., 1999; Dick et al., 2001, 2003; Fujimoto et al., 2002; Haeberle et al., 2004; Nishimune et al., 2004; Shibasaki et al., 2004b; Hagiwara et al., 2005; Khimich et al., 2005; Juranek et al., 2006; Siksou et al., 2007; Limbach et al., 2011; Nishimune, 2012).

The identification and molecular characterization of interacting partners revealed that Piccolo and Bassoon use their multi-domain structure to contribute to various features of presynaptic function (Table 1, Figure 1D). These include assembly of AZ scaffolds, organization of neurotransmitter release machinery, linkage of actin dynamics and endocytosis, maintenance of synapse integrity as well as integration of signaling pathways and synapto-nuclear signaling.

**Table 1:** **Interaction partners of Piccolo and Bassoon**.

Binding site on Piccolo and/or Bassoon	Interaction partner [Uniprot ID (name)]	Description/potential function	Reference	Cellular processes
Pclo, Q-domain, aa 372–491 (rat)—absent in Bsn	Abp1, Actin-binding protein 1/drebrin-like, [Q9JHL4 (DBNL_RAT)]	Links piccolo to the actin cytoskeleton and to endocytic machinery	Fenster et al. (2003)	Actin cytoskeleton dynamics
Pclo, Znf2, aa 1010–1033 (rat)—not tested for Bsn	Pra1, prenylated rab3A acceptor [O35394 (PRAF1_RAT)]	Interaction with SV via rab3 and VAMP2/synaptobrevin-2	Fenster et al. (2000)	Membrane trafficking
Pclo, Znf1 521–582 Pclo, Znf2 1010–1071 Bsn, Znf1 162–225 Bsn, Znf2 459–523 (rat)	Siah1, seven in absentia homolog 1 [Q920M9 (SIAH1_RAT)]	E3 ubiquitin ligase, ubiquitinates SV proteins, component of the ubiquitin-proteasome system.	Waites et al. (2013)	Protein turn-over/degradation	
Bsn aa 1360–1692 (rat)—multiple binding sites, not present in Pclo	Dynein light chains Dlc-1, Dlc-2 [P63170 (DYL1_RAT), Q78P75 (DYL2_RAT)]	Link to dynein motors, (retrograde) transport of Piccolo-Bassoon transport vesicles	Fejtova et al. (2009)	Membrane trafficking
Pclo, aa 2197–2350 (rat)—absent in Bsn	GIT1 [Q9Z272 (GIT1_RAT)]	GTPase-activating protein for ADP ribosylation factor family; regulation of actin cytoskeleton, endocytosis	Kim et al. (2003)	Actin cytoskeleton dynamics, Membrane trafficking
Pclo, aa1980–2553 (rat)—absent in Bsn	Daam1, Dishevelled-associated activator of morphogenesis 1 [(D4ABM3 (D4ABM3_RAT)]	Formin, actin cytoskeleton binding protein	Wagh et al. (2015)	Actin cytoskeleton dynamics
Bsn, aa 1653–2087 (rat) Pclo (homologous region)	CtBP1/BARS [Q9Z2F5 (CTBP1_RAT)], CtBP2/RIBEYE [Q9EQH5 (CTBP2_RAT)]	Anchoring of synaptic ribbons to active zones at ribbon synapses; regulation of gene expression; potential relation to membrane trafficking processes	tom Dieck et al. (2005), Jose et al. (2008), Hübler et al. (2012), and Ivanova et al. (2015)	Synapto-nuclear communication, Membrane trafficking, Scaffolding and Assembly of CAZ core complex
Pclo/Acz, ~aa 2300–2400 PP (mouse) Absent in Bsn	Profilin-2 > profilin-1 [Q9JJV2 (PROF2_MOUSE)]	Actin modulating protein	Wang et al. (1999)	Actin cytoskeleton dynamics
Bsn, CC2 aa 2088–2563 (rat)	Bsn CC2 Pclo CC2	Homo-/heterodimerization region, Golgi-binding domain of Bassoon	Dresbach et al. (2006) and Maas et al. (2012)	Scaffolding and Assembly of CAZ core complex
Pclo/Acz, CC2 aa3094–3218 (mouse)	Bsn	Heterodimerization domain, serves presumably scaffold formation	Wang et al. (2009)	Scaffolding and Assembly of CAZ core complex
Pclo/Acz, CC3 aa 3593–3865 (Mouse)	Munc13 (N-term) [Q62768 (UN13A_RAT)], Potentially via CAST	Priming factor, SV priming	Wang et al. (2009)	Scaffolding and Assembly of CAZ core complex, SV priming
Bsn, CC3 aa 2933–2995 (rat) Bsn, CC3 aa 2873–3077 (mouse) Pclo, CC3 aa 3601–3960 Pclo/Acz, CC3 aa 3657–3715 (mouse)	ERC2/ELKS2/CAST [Q8K3M6 (ERC2_RAT)]	Interaction with CAZ scaffolding proteins. Potentially involved in anchoring synaptic ribbons to the active zone	Takao-Rikitsu et al. (2004), tom Dieck et al. (2005) and Wang et al. (2009)	Scaffolding and Assembly of CAZ core complex
Bsn, Ser2845 (rat)	14–3–3η (and other isoforms) [P68511 (1433F_RAT)]	Phospho-dependent regulation of anchoring of bassoon to CAZ Phosphorylation depends on RSK family	Schröder et al. (2013)	Cellular signaling, Scaffolding and Assembly of CAZ core complex?
Bsn, aa 2869–2899 (rat) Pclo (homologous region)	RIM-BPs (RBP1, RBP2) [Q9JIR0 (RIMB1_RAT); Q9JIR1 (RIMB2_RAT)]	RIM binding proteins, presynaptic scaffold/link to voltage-gated Ca_V_2.1 Ca^2+^ channels. Specificity for Pclo unclear.	Davydova et al. (2014)	Calcium signaling, Regulation of VGCCs, Regulation of exocytosis
Bsn, aa 2715–3263 (rat) Not tested for direct interaction with Pclo	D-Amino Acid Oxidase, DAO [O35078 (OXDA_RAT)]	Enzyme that metabolizes the NMDA receptor co-agonist D-serine. DAO activity significantly inhibited by interaction with Bsn	Popiolek et al. (2011)	Cellular signaling, Regulation of enzymatic activity	
Bsn, aa 3601–3820 (C-term) (mouse)	Munc13 (N-term) [Q62768 (UN13A_RAT)] and RIM [Q99NE5 (RIMS1_MOUSE)]	Interaction with presynaptic scaffolding	Wang et al. (2009)	Scaffolding and Assembly of CAZ core complex, SV priming
Bsn, aa 1886–3938 (C-term) (mouse)	Ca_V_β1b; Ca_V_β4 Q8R3Z5 (CACB1_MOUSE) Q8R0S4 (CACB4_MOUSE)	Interaction and potentially localization of presynaptic voltage-gated Ca_V_2.1 and Ca_V_2.2 Ca^2+^ channels	Chen et al. (2011)	Calcium signaling, Regulation of VGCCs
Bsn aa 3263–3938 (rat) Absent in Pclo	Mover [A8WCF8 (TPRGL_RAT)]	SV protein, negative regulator of synaptic release probability	Kremer et al. (2007), Ahmed et al. (2013), and Korber et al. (2015)	Membrane trafficking? Regulation of exocytosis
Pclo, PDZ, aa 3900–4244 (mouse) Absent in Bsn	Rap guanine nucleotide exchange factor 4/GEFII/Epac 2 [Q9EQZ6 (RPGF4_MOUSE)]	cAMP-dependent exocytosis in pancreas β cells	Fujimoto et al. (2002)	Membrane trafficking, Regulation of exocytosis in pancreatic β-cells
Pclo, C2A, aa 4704–5010 (mouse) Absent in Bsn	RIM2, Rab3 interacting molecule C2A domain Q9EQZ7 (RIMS2_MOUSE)	Presynaptic scaffolding protein involved in synaptic vesicle tethering and priming/interaction detected in exocytosis in pancreas β cells	Fujimoto et al. (2002)	Scaffolding and Assembly of CAZ core complex, SV priming?
Pclo, C2A, aa 4704–5010(mouse) Absent in Bsn	Pclo C2A, [Piccolo/Aczonin] [Q9QYX7 (PCLO_MOUSE)]	Site of Piccolo homo-dimerization/ interaction originally detected in pancreas β cells	Fujimoto et al. (2002) and Garcia et al. (2004)	Scaffolding and Assembly of CAZ core complex
Pclo. C2A, aa 4704–5010 and Pclo, C2B, aa 4955–5165 Absent in Bsn	Ca_V_1.2 [Q01815 (CAC1C_MOUSE)]	L-type voltage gated Ca^2+^ channel/interaction detected in exocytosis in pancreas β cells	Shibasaki et al. (2004a)	Calcium signaling, Regulation of VGCCs

*Abbreviations: Bsn, Bassoon; Pclo, Piccolo; C2A, C2B, C2 domains of Piccolo; CC1–3, coiled-coil regions; PDZ, PDZ domain; PP, proline-rich domain; SV, synaptic vesicles; Znf1–2, zinc finger domains; SV, synaptic vesicle; UniProt information: Piccolo rat: Q9JKS6 (PCLO_RAT); Piccolo/Aczonin mouse: Q9QYX7 (PCLO_MOUSE); Bassoon rat: O88778 (BSN_RAT); Bassoon mouse: O88737 (BSN_MOUSE)*.

## Scaffolding and assembly of Active Zones

The assembly of presynaptic AZs starts at the trans-Golgi network, where components are recruited into precursor vesicles and delivered to nascent synapses (Ahmari et al., 2000; Zhai et al., 2001; Garner et al., 2002; Shapira et al., 2003; Tao-Cheng, 2007; Fairless et al., 2008; Bury and Sabo, 2011; Maas et al., 2012). Although it was initially thought that AZ proteins were pre-assembled on a single precursor vesicle (Ahmari et al., 2000; Zhai et al., 2001; Shapira et al., 2003), more recent studies argue for the existence of at least three types of precursor vesicles that carry complexes between Piccolo-Bassoon-ELKS/CAST, RIM-Neurexin-CASK-voltage-gated Ca^2+^ channels (VGCCs) or Munc13 (Tao-Cheng, 2007; Fairless et al., 2008; Maas et al., 2012). Intriguingly, the assembly of Piccolo-Bassoon-ELKS/CAST transport vesicles (PTV) not only depends on Piccolo and Bassoon, but also on their ability to interact with Golgi-membranes, ELKS/CAST and the membrane fission protein CtBP1/BARS (Dresbach et al., 2003, 2006; Maas et al., 2012). The trafficking and delivery of PTVs to nascent and mature synapses utilizes microtubule-based transport (Fejtova et al., 2009; Bury and Sabo, 2011; Maas et al., 2012) and the activities of anterograde and retrograde motors. Here, attachment to the retrograde motor dynein is mediated by the direct interaction of Bassoon with dynein-light chains (Dlc1/2; Fejtova et al., 2009). Kinesins are also involved and require the adaptor protein Syntabulin, though the linkage to PTVs is currently unknown (Cai et al., 2007).

Once delivered to nascent synapses, the core AZ complex assembles in response to transsynaptic signals from adhesion molecules such as Neurexins and Neuroligins (Waites et al., 2005; Siddiqui and Craig, 2011), occurring within minutes of initial axo-dendritic contact (Vardinon-Friedman et al., 2000; Garner et al., 2002; Lucido et al., 2009). The organization of this complex is determined by a series of protein-protein interactions between Munc13, RIM, Liprin-α, ELKS/CAST, RBP, Bassoon and Piccolo (see Ackermann et al., 2015). From the Bassoon-Piccolo perspective, this can be mediated via domains that promote interactions between Piccolo-Bassoon (coiled-coil region 2, CC2), Bassoon-Piccolo-ELKS/CAST and/or Munc13 (CC3), Piccolo-RIM (C2A domain) or Bassoon-RBPs (Figure 1; Table 1). These are complemented by interactions between Munc13, RIM, ELKS/CAST, Liprin-α and RBPs (Wang et al., 2009; Gundelfinger and Fejtova, 2012; Südhof, 2012). Together, this scaffold is thought to create a platform for integrating other key features of the CAZ.

Importantly, loss of function studies of Bassoon or Piccolo have not been shown to affect the ultra-structural organization of presynaptic AZs of central synapses (Altrock et al., 2003; Leal-Ortiz et al., 2008; Mukherjee et al., 2010), perhaps due to structural redundancy between these and/or other CAZ proteins. Notable exceptions have been observed at vertebrate sensory synapses. Here, loss of Bassoon disrupts the attachment of synaptic ribbons to the arciform density at retinal photoreceptor terminals and at auditory inner hair cell synapses (Dick et al., 2003; Khimich et al., 2005; tom Dieck et al., 2005). At these synapses, Bassoon appears to utilize its interactions with Ribeye and ELKS/CAST (Table 1) to tether ribbons to the AZ (Takao-Rikitsu et al., 2004; tom Dieck et al., 2005; Magupalli et al., 2008). Conversely, loss of the main Piccolo isoform (Piccolino) from mouse photoreceptor cells alters the maturation and ultrastructure of ribbons, i.e., their transition from spherical to ribbon shape structures (Regus-Leidig et al., 2013, 2014).

## Exocytosis and Localization of Voltage-Gated Ca^2+^ Channels

A core function of presynaptic AZs is the regulated release of neurotransmitters, a process which involves the tethering, docking, priming and fusion of SVs with the AZ plasma membrane in a calcium-dependent manner (Südhof, 2012). This requires a delicate interplay between RIMs, Munc13s, SNARE complexes, synaptotagmin and VGCCs. Recent studies on synapses lacking Bassoon indicate a role for this CAZ protein in the recruitment of SVs into vacated release sites as well as the positioning of VGCC near release sites, i.e., positional priming (Frank et al., 2010; Hallermann et al., 2010; Jing et al., 2013; Mendoza Schulz et al., 2014). Regarding the latter, Bassoon, similar to RIMs, has been found to bind RBPs—molecules known to associate with VGCCs (Hibino et al., 2002). In contrast to RIMα-isoforms, which appear to use RBPs to tether various types of VGCCs (e.g., Ca_V_2.2 or N-, and Ca_V_2.1 or P/Q-types) to AZs (Han et al., 2011; Gundelfinger and Fejtova, 2012; Kaeser et al., 2012), Bassoon selectively positions P/Q-type channels near SV release sites of hippocampal synapses (Davydova et al., 2014). At present, it is unclear why different protein interactions are used to tether VGCC into the AZs. One explanation is that it allows synapses to have functional diversity and/or plasticity. For example, N- and P/Q-type channels have been linked to immature and mature synapses, respectively (Scholz and Miller, 1995). Theoretically, the loss of Bassoon and thus P/Q type channels could result in more immature synapses. Based on our present knowledge one can hypothesize that RIMα-isoforms are the key regulators for VGCC recruitment into the AZ, while Bassoon seems to be involved in more subtle subtype-specific regulation of positional priming events at various types of brain synapses (Südhof, 2012; Davydova et al., 2014).

At inner ear hair cell ribbon synapses, Bassoon also contributes to the appropriate localization of L-type VGCC (Ca_V_1.3). Ca^2+^ influx and Ca_V_1.3 density is reduced in *Bassoon (Bsn)*-mutant mice (Khimich et al., 2005; Frank et al., 2010). This phenotype can be partly attributed directly to Bassoon function, but is enhanced by the lack of ribbon anchoring due to loss of Bassoon (Jing et al., 2013).

For the vertebrate neuromuscular junction, the C-terminal third of Bassoon has been shown to interact with β1b and β4 VGCC subunits suggesting an involvement of these interactions in the recruitment, localization and regulation of presynaptic calcium channels (Chen et al., 2011; Nishimune et al., 2012). Additional evidence for an interaction between VGCC and Piccolo and/or Bassoon derives from the proteomic analysis of calcium channel complexes both in the CNS (Müller et al., 2010) and at the neuromuscular junction (Carlson et al., 2010).

In contrast to Bassoon, a functional link between Piccolo and the localization of VGCC at brain synapses is less well explored. Here, one point of focus with respect to the regulated neurotransmitter release has been on its C2A domain. Similar to other C2A domains, this region in Piccolo binds calcium (albeit with low affinity) and induces a conformational change in this domain (Gerber et al., 2001; Garcia et al., 2004) that affects its association with phospholipids and its dimerization. However, no effect on synaptic transmission was observed if mutations that disrupt calcium binding were knocked into the C2A domain in the *Piccolo (Pclo)* gene (Mukherjee et al., 2010), leaving open the role of the C2A domain in CNS synapse function. Intriguingly, the Piccolo C2A domain has been reported to regulate the surface levels of dopamine transporters (DAT; Cen et al., 2008), and when over-expressed in transgenic mice to induce depression-like behaviors (Furukawa-Hibi et al., 2010), implying a role for Piccolo in dopaminergic transmission. Consistently, a genome wide association study identified a mutation in the C2A domain of Piccolo in patients with major depressive disorders (Giniatullina et al., 2015), though how this mutation causes depression remains unclear.

Other insights into Piccolo’s role in regulated secretion have come from experiments on β-islet cells. Here, the C2A domain was found to bind RIM2 and L-type VGCC (Ca_V_1.2). Moreover, Piccolo can interact with the cAMP-sensor Epac2 (Rap guanine nucleotide exchange factor 4 or cAMP-GEFII; see Table 1) and loss of Piccolo function was found to impair* cAMP-dependent* insulin secretion (Fujimoto et al., 2002; Shibasaki et al., 2004a; Jacobo et al., 2009).

Bassoon and Piccolo seem to have functions in organizing the neurotransmitter release machinery including the tethering and priming of SVs at AZs. Thus both proteins participate in the formation of the CAZ core complex that recruits factors essential for tethering and priming of SVs, such as Munc13s and RIM1/2α-isoforms (Wang et al., 2009; Table 1). Upon over-expression, the Piccolo/Aczonin CC3 region is targeted to nerve terminals and impairs SV recycling similar to the over-expression of the RIM1 zinc finger (Znf) domain. In addition the C-terminus of Bassoon can interact with the N-terminal C2A domain in Munc13 (Wang et al., 2009). It is thus conceivable that this complex interplay might contribute to the regulation of RIM-dependent activation of Munc13 priming functions (Deng et al., 2011; Han et al., 2011; Südhof, 2012).

Mover, a SV-associated phospho-protein, was originally identified as a binding partner for Bassoon (Kremer et al., 2007; Ahmed et al., 2013) and recently characterized as a negative regulator of evoked SV exocytosis (Korber et al., 2015). ShRNA-mediated knock-down of the protein in the Calyx of Held leads to an accelerated and enhanced synaptic depression caused by an increased sensitivity of the release apparatus to Ca^2+^. The role of Bassoon in this context is yet unclear, as a similar synaptic depression phenotypes have not been observed in *Bsn*-mutant mice (Altrock et al., 2003).

Indirect evidence that Bassoon may also be involved in the release of brain-derived neurotrophic factor (BDNF) from large dense-core vesicles comes from the investigation of *Bsn*-mutant mice. Accumulation of high amounts of BDNF, in particular brain regions including hippocampus, cerebral cortex and striatum, has been observed in mutant brains (Ghiglieri et al., 2010; Heyden et al., 2011; Dieni et al., 2012, 2015).

## Actin Dynamics and Endocytosis

While Piccolo and Bassoon share a great deal of structural homology, each also contains unique segments, implying divergent functions. Studies of Piccolo have shown that it is selectively required for activity-induced F-actin assembly within presynaptic boutons and efficient synaptic transmission (Waites et al., 2011). Consistent with this concept, Piccolo, but not Bassoon, contains regions that interact with a variety of actin-associated proteins (Figure 1D; Table 1). These include Abp1 (Fenster et al., 2003), GIT1 (Kim et al., 2003), Daam1 (Wagh et al., 2015), Profilin2 (Wang et al., 1999; Waites et al., 2011) and Epac2 (Fujimoto et al., 2002). Functionally, these interactions appear to contribute to the regulated delivery and recycling of SVs within nerve terminals. For example, emerging evidence indicate that Profilin2, GIT1 and Daam1 may contribute to the polymerization of linear rather than branched F-actin filaments from AZs (Wagh et al., 2015), thought to be involved in the translocation of SVs from the reserve into the readily releasable pools. In this regard, Piccolo seems to act as a platform for the polymerization of these filaments via a region situated between CC1 and CC2 (Wagh et al., 2015). This region binds to GIT1, a GTPase-activating protein (GAP) for ADP-ribosylation factors (ARFs), involved in the regulation of membrane trafficking (Chavrier and Goud, 1999), as well as PIX1, a focal adhesion kinase, and Paxillin, both of which regulate the assembly of the actin cytoskeleton at focal adhesion site (Kim et al., 2003). This region also binds Daam1, a member of the formin family of molecules that directly polymerize actin upon activation by RhoA and/or the Wnt/Disheveled signaling complex (Habas et al., 2001; Liu et al., 2008) and Profilin2, an ADP/ATP nucleotide exchange factor for G-actin (Kovar, 2006), that binds at the mouth of formins, allowing for the rapid processive assembly of linear F-actin polymer (Higgs, 2005). Importantly, Daam1 only binds Piccolo and promotes F-actin assembly after activation, implying that Piccolo spatially controls the assembly of these filaments radial out from the AZ.

Intriguingly, two of these Piccolo binding partners, Abp1 and GIT1 are also linked to SV endocytosis (Fenster et al., 2003; Kim et al., 2003). For example, the actin-binding protein, Abp1 has been shown to interact the endocytic protein Dynamin (Kessels et al., 2001), while GIT1 directly associates with Stonin2, an endocytic adaptor protein (Podufall et al., 2014). In flies, SV recycling is impaired at synapses lacking GIT1 and is associated with the displacement of Stonin2/StonedB away from AZ implying that GIT1, and possibly Piccolo in vertebrates, help to couple exo- and endocytosis by creating a bridge between active and endocytic zones (Podufall et al., 2014). Of note, ultrafast endocytosis and bulk endocytosis both require the dynamic assembly of F-actin (Akbergenova and Bykhovskaia, 2009; Nguyen et al., 2012; Watanabe et al., 2013a,b), a process that could be facilitated via complexes between Piccolo, GIT1, Abp1 and other F-actin assembly proteins at endocytic sites.

## Synapse Maintenance and integrity

In addition to their roles in the structural and functional assembly of presynaptic AZs, Piccolo and Bassoon are involved in the maintenance of SV pools (Mukherjee et al., 2010) and act as regulators of presynaptic proteostasis and the integrity of synapses as observed upon knock-down of the proteins in primary neuronal culture (Waites et al., 2013). This new functionalities were only revealed at synapses lacking both proteins implying that it involves structurally conserved regions between these molecules. In this regard, there seem to be three mechanisms that contribute to the stability and integrity of presynaptic AZs. The first involves the dynamic exchange of synaptic proteins, which occurs on the order of tens of minutes to hours (Minerbi et al., 2009; Ziv and Fisher-Lavie, 2014); the second one the activity-dependent reorganization of AZs, which modulate the efficiency of neurotransmitter release (Lazarevic et al., 2011, 2013); and the third one the turnover/degradation of synaptic proteins, most of which have half-lives of 2–7 days (Cohen et al., 2013). Regarding the latter, Piccolo and Bassoon appear to play fundamental roles in regulating the ubiquitin-proteasome (UPS) and autophagy-lysosomal systems within presynaptic boutons (Waites et al., 2013). Specifically, it was observed that, in boutons lacking these AZ molecules, SV pools and synaptic junctions were lost. Moreover, ubiquitinated proteins, pleomorphic vesicles, and multi-vesicular bodies were observed to accumulate within these degenerating boutons, a process that required the activation of the UPS, lysosomal and ubiquitin systems. Thus far one molecular link between Piccolo and Bassoon and these degradative systems has been identified, i.e., Siah1 (Waites et al., 2013). Siah1 is an E3 ubiquitin ligase involved in the poly-ubiquitination of SV proteins such as synaptophysin and synuclein. Importantly, Siah1 binding to the Znf domains of Bassoon and Piccolo inhibits its activity (Waites et al., 2013). The functional importance of this locally regulated degradative system is unclear. Possibilities include the regulated release of neurotransmitter, the recycling of SVs and/or the removal of misfolded or aging proteins (Waites and Garner, 2011; Ackermann et al., 2015).

## Presynapse-to-Nucleus Signaling

Communication between synapses and the soma, in particular the nucleus, of a neuron is essential for neuronal survival as well as for processes of homeostatic and associative plasticity. While multiple synapto-nuclear signaling pathways have been reported for the postsynapse (for review, see Jordan and Kreutz, 2009; Panayotis et al., 2015), much less is understood about presynapse to nucleus communication. Known pathways from the presynapse or the axon to the nucleus include retrograde signaling of neurotrophins via signaling endosomes (Cosker and Segal, 2014) and retrograde signal transduction from sites of injury (Panayotis et al., 2015). One recently discovered signaling pathway involves Bassoon and Piccolo as synaptic key regulators for presynaptic recruitment and release of the transcriptional co-repressor CtBP1 (aka BARS; Ivanova et al., 2015). The interaction between Bassoon or Piccolo and CtBP1 is mediated by the metabolic sensor system NAD^+^/NADH and contributes to activity-dependent distribution of CtBP1 between presynapses and the nucleus. High activity shifts the equilibrium between synaptic and nuclear CtBP1 pools to synapses, low activity to the nucleus (Ivanova et al., 2015; Kravchick and Jordan, 2015). CtBP1 (as its paralogue CtBP2) is involved both gene-specific and global repression of gene transcription and has been implicated in processes of development and tumorigenesis (Chinnadurai, 2007). By controlling the synapto-nuclear distribution of CtBPs, Bassoon and Piccolo contribute significantly to the adjustment of activity-regulated gene expression and in turn to processes of long-term implementation of memories.

Of note, the interaction of CtBPs with Bassoon and Piccolo has long been known (tom Dieck et al., 2005; Jose et al., 2008; Hübler et al., 2012). Because of its functional involvement in dynamin-independent fission events, such as budding of vesicles from trans-Golgi membranes, macropinocytosis or fluid-phase endocytosis (Corda et al., 2006; Valente et al., 2013) the interaction may play also a role in membrane trafficking events within the presynapse and from the Golgi to the synapse (e.g., Maas et al., 2012). Moreover, as mentioned above, Bassoon’s interaction with an N-terminally extended isoform of CtBP2, i.e., Ribeye (Schmitz et al., 2000), plays an important role in the assembly and anchoring of synaptic ribbons to their AZs in retinal photoreceptors and inner ear hair cells (Khimich et al., 2005; tom Dieck et al., 2005; Magupalli et al., 2008). An important question is how CtBPs act in so many different cellular functions? One simple concept is that it works together with different scaffold proteins such as Bassoon and Piccolo to direct its activity as an NAD/NADH sensor to control vesicular membrane fission, synaptic ribbon assembly and nuclear gene expression.

## Conclusions

The detailed analysis of protein binding partners for Piccolo and Bassoon support the concept that they are fundamentally involved in scaffolding a large number of proteins involved with various aspect of presynaptic function within AZs. State of the art *in silico* analysis of their structure (Figure 1D) reveals that individual subdomains of these molecules are likely organized into larger modules, which might facilitate the assembly of supera-molecular complexes devoted to specific AZ functions. For example, the C-terminal halves of both molecules seem to organize proteins involved in the docking, molecular priming and positional priming of SVs. Conversely the central regions of these molecules possess modules that have acquired unique functions, e.g., devoted to the dynamic assembly of actin and endocytosis vs. vesicular transport for Piccolo and Bassoon, respectively. Clearly the relevance of these super-modules needs to be explored further. Finally, although not discussed here, the functional relevance of the large number of posttranslational modifications (phosphorylation, O-glycosylation, ubiquitination; Trinidad et al., 2006; Vosseller et al., 2006; Munton et al., 2007; Chalkley et al., 2009; Lazarevic et al., 2011; Schröder et al., 2013; Ackermann et al., 2015) has yet to be examined, though roles in the regulated rearrangement during learning-related plasticity of AZs are likely (e.g., Kähne et al., 2012).

## Author Contributions

EDG and CCG have written the article. CR contributed essential discussions and designed Figure 1D.

## Conflict of Interest Statement

The authors declare that the research was conducted in the absence of any commercial or financial relationships that could be construed as a potential conflict of interest.
